# Production of Organic Acids by Arbuscular Mycorrhizal Fungi and Their Contribution in the Mobilization of Phosphorus Bound to Iron Oxides

**DOI:** 10.3389/fpls.2021.661842

**Published:** 2021-07-15

**Authors:** Alberto Andrino, Georg Guggenberger, Sarmite Kernchen, Robert Mikutta, Leopold Sauheitl, Jens Boy

**Affiliations:** ^1^Institute of Soil Science, Leibniz Universität Hannover, Hanover, Germany; ^2^Bayreuth Center of Ecology and Environmental Research, University of Bayreuth, Bayreuth, Germany; ^3^Soil Science and Soil Protection, Martin Luther University Halle-Wittenberg, Halle (Saale), Germany

**Keywords:** arbuscular mycorrhiza, low-molecular-weight organic acid, iron oxides, membrane fluidity, organic P, inorganic P, ligand exchange, reductive dissolution

## Abstract

Most plants living in tropical acid soils depend on the arbuscular mycorrhizal (AM) symbiosis for mobilizing low-accessible phosphorus (P), due to its strong bonding by iron (Fe) oxides. The roots release low-molecular-weight organic acids (LMWOAs) as a mechanism to increase soil P availability by ligand exchange or dissolution. However, little is known on the LMWOA production by AM fungi (AMF), since most studies conducted on AM plants do not discriminate on the LMWOA origin. This study aimed to determine whether AMF release significant amounts of LMWOAs to liberate P bound to Fe oxides, which is otherwise unavailable for the plant. *Solanum lycopersicum* L. plants mycorrhized with *Rhizophagus irregularis* were placed in a bicompartmental mesocosm, with P sources only accessible by AMF. Fingerprinting of LMWOAs in compartments containing free and goethite-bound orthophosphate (OP or GOE-OP) and phytic acid (PA or GOE-PA) was done. To assess P mobilization *via* AM symbiosis, P content, photosynthesis, and the degree of mycorrhization were determined in the plant; whereas, AM hyphae abundance was determined using lipid biomarkers. The results showing a higher shoot P content, along with a lower N:P ratio and a higher photosynthetic capacity, may be indicative of a higher photosynthetic P-use efficiency, when AM plants mobilized P from less-accessible sources. The presence of mono-, di-, and tricarboxylic LMWOAs in compartments containing OP or GOE-OP and phytic acid (PA or GOE-PA) points toward the occurrence of reductive dissolution and ligand exchange/dissolution reactions. Furthermore, hyphae grown in goethite loaded with OP and PA exhibited an increased content of unsaturated lipids, pointing to an increased membrane fluidity in order to maintain optimal hyphal functionality and facilitate the incorporation of P. Our results underpin the centrality of AM symbiosis in soil biogeochemical processes, by highlighting the ability of the AMF and accompanying microbiota in releasing significant amounts of LMWOAs to mobilize P bound to Fe oxides.

## Introduction

Phosphorus (P) is an essential plant macronutrient (Schachtman et al., 1998), and its deficiency limits the plant growth in both natural and agricultural systems (Oberson et al., 2001). Particularly, in acidic soils, the high affinity and strong specific adsorption of inorganic (Pi) and organic (Po) phosphorus forms to iron (Fe) oxides determine their accessibility to plants (He and Zhu, 1998). Rhizosphere acidification and the release of low-molecular-weight organic acids (LMWOAs) are the important plant response mechanisms to increase P availability in the soil solution (Wang et al., 2019). The LMWOAs may solubilize P from mineral surfaces either by ligand exchange or by ligand-promoted dissolution of Fe oxides (Owen et al., 2015). The ability of different LMWOAs to desorb P generally decreases with a decrease in the stability constants of Fe (III) acid complexes (Marschener, 1998; Deubel and Merbach, 2005). The adsorption of LMWOAs is driven by positively charged oxide surfaces and the negative charge of the carboxylate group and is influenced by the formation of metal complexes in solution, with adsorption generally increasing with their concentration in solution and the number of carboxylic groups (Oburger et al., 2011; Adeleke et al., 2017). Thus, tricarboxylic acids such as citrate have a higher efficiency to desorb P from Fe oxides than dicarboxylic or monocarboxylic ones (Geelhoed et al., 1999; Richardson, 2001).

The association of plants with symbiont organisms is one of the most widespread strategies employed to mobilize P in acidic tropical soils (Seguel et al., 2013). In particular, the association with arbuscular mycorrhizal fungi (AMF) is central to the P cycling, mobilization, and supply to plants adapted to acidic environments (Klugh and Cumming, 2007). The arbuscular mycorrhizal (AM) symbiosis promotes the formation of an extensive mycelium network that operates as functional extensions of the plant root system (Xu et al., 2007), exchanging the acquired P for fresh assimilated photosynthetic carbon (C) from the host plant (Zhang et al., 2016). Furthermore, AMF may act as hub translocating freshly assimilated C to soil microbes on the surfaces of mycorrhizal hyphae, spores, and the hyphosphere, the zone surrounding individual fungal hyphae (Zhang et al., 2014; Manchanda et al., 2017). The accompanying AMF microbiota may be functionally diverse and provide essential plant growth-promoting functions, such as phytate mineralization, siderophore production, Pi solubilization, and LMWOA production (Battini et al., 2016). In this way, the association of AMF with bacteria provides a beneficial partnership for accessing and mobilizing soil P pools, which otherwise would not be available to the plant (Wang et al., 2016; Drigo and Donn, 2017). Phosphorus mobilization by AMF may involve both Pi (Smith and Read, 2008) and Po forms (Andrino et al., 2020). There is also evidence that AMF can desorb OP from ferrihydrite (Gogala et al., 1995; Rakshit and Bhadoria, 2010), and recently, we confirmed the ability of *R. irregularis* to mobilize Po and Pi bound to goethite (GOE), one of the most abundant Fe (oxy)hydroxides in tropical soils, at differing host plant C cost (Andrino et al., 2019).

The release of P bound to pedogenic oxides requires the action of LMWOAs (Geelhoed et al., 1999), but the production of LMWOAs by AMF is still poorly documented (Bharadwaj et al., 2012). Sato et al. (2015) and Burghelea et al. (2018) pointed out that AMF exudates involved in P mobilization from Po and Pi sources may comprise phosphatases, phenolic compounds, protons, siderophores, and an increased root exudation of organic ligands; however, studies on the production of LMWOAs by AMF are scarce (Tawaraya et al., 2006; Toljander et al., 2007). Consequently, the present study seeks to understand the role of LMWOAs secreted by the AMF to the P mobilization from GOE-bound P sources. We hypothesize that the suite of LMWOAs produced when mobilizing P from GOE-bound orthophosphate (OP or GOE-OP) and phytic acid (PA or GOE-PA) sources differs from those in the presence of their free P forms, as a consequence of ligand dissolution processes. To this end, we used a bicompartmental mesocosm consisting of a plant compartment (PC) harboring one *Solanum lycopersicum* L. plant mycorrhized with *Rhizophagus irregularis*; however, the fungal compartment (FC) contained free or OP or GOE-OP and PA or GOE-PA only accessible by the AM fungus. To assess P mobilization via AM symbiosis, P and N contents, photosynthesis, and the degree of mycorrhization were determined in the plant; however, LMWOAs fingerprint and the AM hyphae abundance were determined using the FC.

## Materials and Methods

### Phosphorus Sources

Four P sources were prepared to be added individually into the FCs as described in Table 1. OP was added as KH_2_PO_4_ (Sigma-Aldrich, Steinheim, Germany), whereas PA was added as sodium salt (Sigma-Aldrich, Steinheim, Germany). The adsorption complexes were prepared by equilibrating P compounds with GOE (Bayferrox 920 Z. Lanxess, Cologne, Germany). The first step involved the equilibration of 50 g of GOE for 16 h in 250 ml ultrapure water adjusted to pH 4 by 0.5 M HCl. Second, 250 ml ultrapure water containing either 17 g KH_2_PO_4_ or 0.72 g C_6_H_18_O_24_P_6_ and adjusted to pH 4 by 0.5 M HCl was added to the GOE solution and equilibrated for 48 h on an overhead shaker. The GOE-P suspensions were centrifuged for 15 min (3,000 × *g*), and pellets were afterward rinsed with ultrapure water until the electric conductivity was <40 μS cm^–1^. Finally, the resulting GOE-P associations were shock-frozen in liquid N_2_ and freeze-dried. The loading of OP and PA onto the GOE was determined by hydrolyzing 5 mg of the GOE-P associations in concentrated HNO_3_ (*n* = 3) and subsequent measurement of P contents by ICP-MS Agilent 7500C (Agilent Technologies, Santa Clara, CA, United States). The adsorption complexes contained 1.24 mg P g^–1^ for GOE-OP and 1.79 mg P g^–1^ for GOE-PA.

**TABLE 1:** Description of the treatments tested during the time course experiment.

**Codes**	**Treatment**	**Phosphorus content at the fungal compartment**
M+	Control: treatment containing an arbuscular mycorrhizal (AM) plant and no phosphorus (P) source in the fungal compartment (FC)	Quartz sand (60 g) + MilliQ water (16 ml) containing no P
M-	Control: non-AM plant and no P source in the FC	Quartz sand (60 g) + MilliQ water (16 ml) containing no P
GOE	Control: AM plant and no P source in the FC	Bayferrox 920 Z goethite (24.3 g) + MilliQ water (28ml) containing no P
OP	AM plant and orthophosphate (KH_2_PO_4_) as P source in the FC	Quartz sand (60 g) + containing 30 mg P (16 ml)
PA	AM plant and phytic acid solution (C_6_H_18_O_24_P_6_⋅xNa^+^⋅yH_2_O) as P source in the FC	Quartz sand (60 g) + containing 30 mg P (16 ml)
GOE-PA	AM plant and phytic acid bound to goethite adsorption complex (1.79 g P/kg) as P source in the FC	GOE-PA (16.7 g) containing 30 mg P + Bayferrox 920 Z goethite (7.6 g) + MilliQ water (28 ml)
GOE-OP	AM plant and orthophosphate bound to goethite adsorption complex (1.24 g P/kg) as P source in the FC	GOE-OP (24.3 g) containing 30 mg P + MilliQ water (28 ml)

### Plant Mycorrhization

*Solanum lycopersicum* L. var. Moneymaker seeds (Volmary GmbH) were surface-sterilized (5% H_2_O_2_, 10 min), soaked in distilled and autoclaved water, and pregerminated on petri dishes (72 h, 27°C). We selected *R. irregularis* DAOM 197198 as AMF due to its global distribution and well adaptation to intensive agricultural practices (Köhl et al., 2016). The inoculum consisted of 0.4 g containing AMF propagules (roots, spores, hyphae) of *Sorghum bicolor* inoculated with *R. irregularis* DAOM 197198 (Symplanta GmbH & Co. KG. Darmstadt, Germany) grown in a trap plant culture following the methodology of Brundrett et al. (1996). The combination of both organisms has been selected in several other research studies as a model of mutualistic association (Herrera-Medina et al., 2008; Fernández et al., 2014). Tomato pregerminated seeds were planted in QP96 cells (HerkuPlast Kubern GmbH, Ering, Germany) together with the inoculum of *R. irregularis* and 70 ml of autoclaved and acid-washed quartz sand. The quartz sand was used as a nutrient-free culture substrate suitable for the colonization of AMF (Table 1), where high-purity mycelium can develop (Johansen et al., 1996; Olsson and Johansen, 2000). For the non-mycorrhizal plants, we grew *S. bicolor* without including any AMF inoculum. Then, 0.4 g of non-inoculated inoculum was applied after checking that no endophyte was colonizing the roots. Mycorrhized and non-mycorrhized tomato plants were grown in a greenhouse (photoperiod, 16/8 h light/dark; temperature, 24/20°C light/dark; relative humidity, 50–60%; photon flux density, 175–230 μmol m^–2^ s^–1^). *S. lycopersicum* seedlings were watered every day with 10 ml deionized water and on alternate days were fertilized with 5 ml low P (0.32 mM) modified Long Ashton nutrient solution pH 6.5 (Hewitt, 1966).

### Time Course Experiment

**FIGURE 1:**
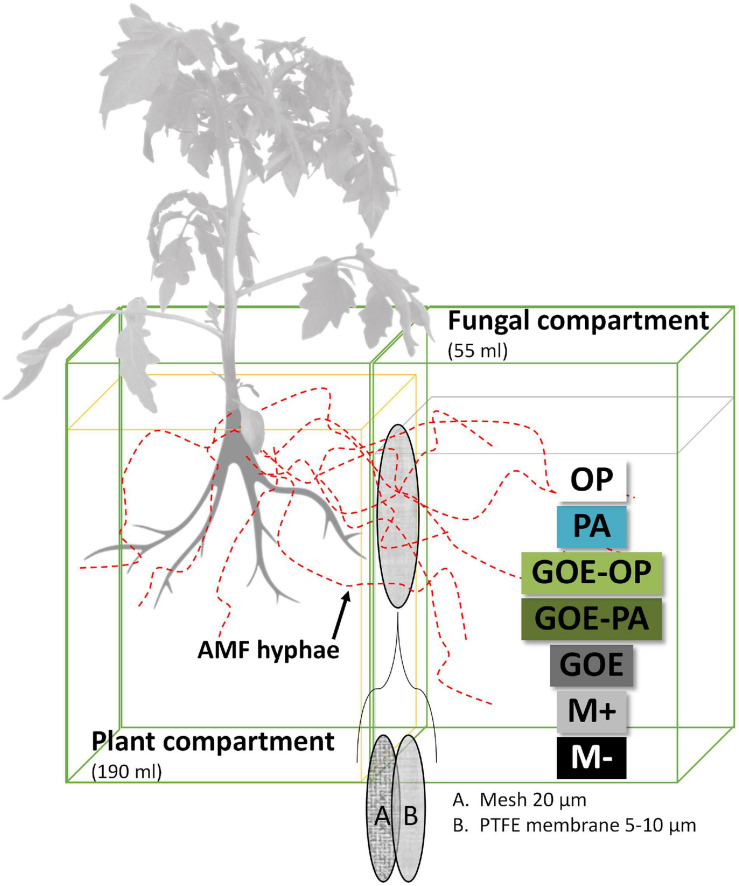
Scheme of the bicompartmental mesocosms, comprising a plant and a fungal compartment. A polyamide mesh (20 μm pore diameter) separated mycorrhizal roots and mycelium, while the polytetrafluoroethylene (PTFE) membrane (5–10 μm pore diameter) allowed the AMF to cross and access the individual P sources but avoided the diffusion of ions into the plant compartment. OP, orthophosphate; PA, phytic acid; GOE-PA, phytic acid bound to goethite; GOE-OP, orthophosphate bound to goethite; GOE, mycorrhized plant, no P, and goethite; M+, mycorrhized plant, no P, quartz sand; M-, non-mycorrhized plant, no P, quartz sand.

The mesocosms were made of two compartments, a PC and a FC. In the latter, exclusively hyphae could enter and access the four P sources (Figure 1), as a polyamide mesh (20 μm pore diameter) (Franz Eckert GmbH, Waldkirch, Germany) separated mycorrhizal roots and mycelium (Watkins et al., 1996; Fitter et al., 1998). A second polytetrafluoroethylene membrane (5–10 μm pore diameter) (Pieper Filter GmbH, Bad Zwischenahn, Germany) allowed the AMF hyphae to cross but avoided the diffusion of ions into the PC (Mäder et al., 2000). The different P sources were placed into the FC as described in Table 1. Three types of controls were included, one without a mycorrhized plant and without a P source, to evaluate how the tomato plant responds to the absence of P (M-): two more controls both with mycorrhized plants and without P, one containing quartz sand (M+) and one with only GOE in the FC (GOE), to the evaluate possible effects related to the substrate where the fungus grows, but not to the P source (Table 1). Four-week-old mycorrhizal and control *S. lycopersicum* plants were planted into the PC. Mesocosms, comprising three biological replicates per P source and harvest point, were placed in a climatic controlled greenhouse (photoperiod, 16/8 h light/dark; temperature, 24/20°C light/dark; relative humidity, 50–60%; photon flux density, 175–230 μmol m^–2^ s^–1^). They were watered two times a week with 10 ml ultrapure water. On alternate days, the pots were fertilized with 5 ml no-P Long Ashton nutrient solution. Once a week, the mesocosms were rotated to achieve homogeneous growth conditions for all mesocosms. The first sampling point was at the day of transplanting (day 0) to determine the initial plant biomass, P and N contents, and photosynthetic activities (*n* = 5) followed by harvest points at days 7, 21, 35, 49, 77, and 91.

### Plant Biomass, Phosphorus, and Nitrogen Contents

At each harvest point, shoots and roots were dried (70°C, 48 h), weighed, and ball-milled. Aliquots of shoot and root were incinerated at 480°C for 8 h, digested in 1 ml 30% HNO_3_, and filtered to <0.45 μm (PVDF filters), and the solutions were analyzed for P content by ICP-MS Agilent 7500C (Agilent Technologies, Santa Clara, CA, United States). Total N content from the milled shoot material was measured by dry combustion using an Elementar vario MICRO cube C/N Analyzer (Elementar GmbH, Hanau, Germany). Shoot and root P contents in percentage of total plant dry weight (% dw) were calculated for each sampling point and treatment. Total P acquired into the plant tissues was calculated by subtracting the total P content (mg) in a subsample (*n* = 5) of the initial transplanted AM plants at day 0 from the total P content (mg) at each harvest point. The shoot N:P ratio, an indicator for P deficiency in the shoot tissues (Hayes et al., 2014; Ros et al., 2018), was calculated for plants accessing the different P sources and controls. Shoot N:P ratios are useful to investigate shifts from N to P limitation because they are easily determined and comparable across studies. Nitrogen limitation for terrestrial plants occurs at values below 10, while P limitation usually occurs above 20 and may cause the inhibition of photosynthesis (Güsewell, 2004).

### Photosynthetic Capacity

At each harvest point, the photosynthetic capacity (μmol CO_2_ m^–2^ s^–1^) was measured on recently fully expanded third or fourth leaf from the top, in order to check the impact of the different P sources on the host carbohydrate metabolism. At each sampling point, CO_2_ assimilation rate was measured with the LI-6400 (LiCor, Lincoln, NE, United States). Values were recorded at 22°C in the leaf cuvette, at approximately 50% relative humidity, airflow rate was set at 400 μmol/s, the external CO_2_ concentration was 360 ppm, and the CO_2_ mixer was set at 400 ppm. Irradiance was provided by a led source set to a photon flux density of 1,000 μmol m^–2^ s^–1^.

### Degree of Mycorrhization

Before planting the seedlings into the mesocosms and at each harvest point, a root subsample was digested in 10% KOH (35 min, 95°C) and stained using the ink and vinegar staining technique for AMF of Vierheilig et al. (1998). Then, the stained root fragments were arranged on microscope slides with fine tweezers, and the degree of mycorrhization was determined using the methodology of McGonigle et al. (1990). In brief, number of arbuscules, vesicles, and internal hyphae were counted using a compound microscope with an eye piece cross-hair, which is moved to randomly selected positions. Arbuscules, vesicles, and total mycorrhization were expressed as the percentage of the total counted intersections.

### Low-Molecular-Weight Organic Acids

Concentration and composition of LMWOAs in the FC containing the different P sources were analyzed in order to determine their role in P acquisition from the different sources. For each treatment containing a P source, the mean content of each LMWOA was calculated using the dates of the harvesting points for the periods where no P incorporation was detected in the AM plant tissue, as well as for those where we detected P uptake in the AM plant. LMWOAs were determined by the method of Tani and Higashi (1999). In brief, 5 g moist sample of each FC was extracted with NH_4_-phosphate buffer (0.1 M NH_4_H_2_PO_4_–H_3_PO_4_, pH 2) at a sample (related to dry sample mass) to solution ratio of 1 (wt.):4 (vol), by shaking for 30 min on a horizontal shaker. Then, the crude extract was separated from the sample by centrifuging at 10,000 × *g* for 10 min, followed by further filtration through a 0.025-μm filter (Supor^®^ PES membrane disk filters, Pall Life Sciences, Hampshire, United Kingdom). Filtered extracts were analyzed with an Agilent series 1100 liquid chromatograph (Agilent Technologies, Santa Clara, CA, United States) coupled to electrospray ionization (ESI) mass spectrometer (Agilent 6130 single quadrupole) to determine the different LMWOAs. Further details on the methods to analyze the LMWOAs can be found in the Supplementary Material 1.

### Fatty Acid Analysis in the FC

The AMF *R. irregularis* DAOM 197198 has a fatty acid composition ranging from C16:0 to C22:2 (Wewer et al., 2014). The fungal phospholipid fatty acids (PLFAs) 16:1ω5, 18:1ω7c, 18:1ω9c, and 18:2ω6,9 were used as indicators for evaluating the amount of AMF extraradical mycelia, while neutral lipid fatty acids (NLFAs) 16:1ω5, 18:1ω7c, 18:1ω9c, and 18:2ω6,9 signatures were considered as indicators on energy storage by the fungus (Olsson and Wilhelmsson, 2000; Bååth, 2003; van Aarle and Olsson, 2003). Lipids were extracted from 8 g or 16 g fresh weight samples of the FCs containing goethite (GOE, GOE-OP, and GOE-PA) or the ones with quartz (M+, M-, OP, and PA), respectively. Then, extracts were fractionated into PLFA and NLFA by the solid-phase extraction with activated silica gel (Sigma Aldrich, pore size 60 Å, 70–230 mesh). Thereafter, the PLFA and NLFA fractions were saponified into fatty acids, and both types of lipids were esterified with methanol to free fatty acid methyl esters, as outlined in Frostegård et al. (1991) and with modifications by Bischoff et al. (2016). The fatty acid methyl esters were then separated by gas chromatography using an Agilent 7890A GC system (Agilent Technologies, Santa Clara, CA, United States) equipped with a Zebron capillary GC column (60 m, 0.25 mm diameter and 0.25 μm film thickness; Phenomenex, Torrance, California, United States) and quantified with a flame ionization detector, using He as carrier gas. Glyceryl tridodecanoate and non-adecanoic acid were used as internal standards during the extraction, and tridecanoic acid methyl ester was added to each sample and standard before the analysis as a recovery standard. At each time point, the relative abundance (%) of fungal biomarkers (PLFA and NLFA) was calculated for each treatment containing a P source.

### Data Analysis

Data for shoot P contents, root P contents, shoot N:P ratios, photosynthetic activities, and LMWOAs contents were tested for normality with Shapiro–Wilk’s test and homogeneity of variances using the Levene’s test, and the different variables were subjected to one-way ANOVA. The Duncan *post hoc* test was employed to check for differences of mean values (*p* < 0.05) between the different P sources offered in the FC at each sampling point. Moreover, two correlation tests (*p* < 0.05) were carried out: first, between the relative abundance of all detected fungal PLFA biomarkers in the FC and the amount of P acquired by the plant (mg), to assess the link between AMF presence and plant P allocation. In a second correlation, the degree of mycorrhization (arbuscules, vesicles, and mycorrhization), i.e., a proxy for AM activity (McCormack and Iversen, 2019), was related to the relative abundance of fungal biomarkers (PLFA and NLFA). This second correlation intends to determine whether P incorporation into the plant was linked to mycorrhizal root activity, and this was in turn linked to the development of the fungal symbiont inside the FC. The ANOVA and correlation tests were performed with SPSS v. 24 (IBM Corporation, 2016).

## Results

### Phosphorus Contents in the Plant Tissues and Photosynthetic Capacity

**FIGURE 2:**
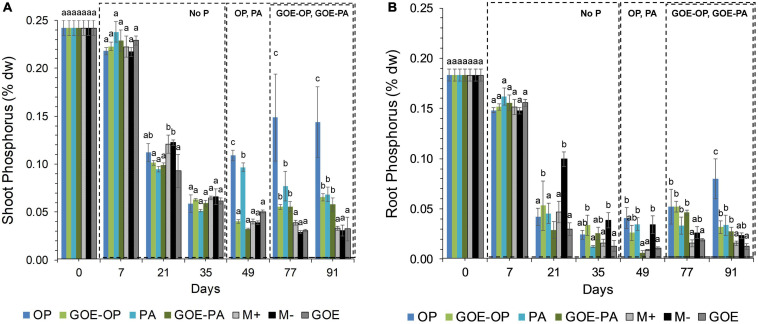
Mean values and standard errors for shoot **(A)** and root **(B)** P content (% dry weight) of *Solanum lycopersicum* L. for the different available P sources and controls during the time course experiment. Three explicative boxes are included to differentiate the periods when we detected the AM plants acquired P from their respective sources. Within each P source and day, treatments with significant differences are labeled with different letters (*p* < 0.05) as result of a one-way ANOVA. OP, orthophosphate; PA, phytic acid; GOE-PA, phytic acid bound to goethite; GOE-OP, orthophosphate bound to goethite; GOE, mycorrhized plant, no P, and goethite; M+, mycorrhized plant, no P, quartz sand; M-, non-mycorrhized plant, no P, quartz sand.

All AM plants with access to a P source in the FC showed significantly larger P contents in the shoots compared to the roots (Figure 2). AM plants that accessed OP and PA or GOE-OP and GOE-PA showed a P dilution in their shoot and root tissues until day 35 or 49, then from day 49 or 77 onward, the P content in their plant tissues significantly increased, respectively, as compared to all controls (Figure 2).

**FIGURE 3:**
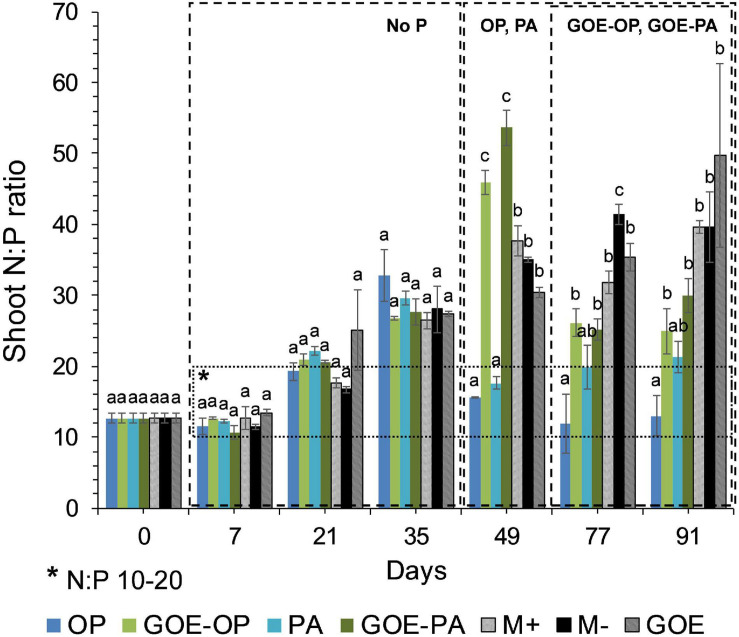
Mean values and standard errors for shoot N:P ratio of *Solanum lycopersicum* L. for the different available P sources and controls during the time course experiment. A box has been drawn covering the values 10–20 of the N:P ratio, to represent the range of the ratio where no nitrogen (<10) or phosphorus (>20) deficiency would exist. Three explicative boxes are included to differentiate the periods when we detected the AM plants acquired P from their respective sources. Within each P source and day, treatments with significant differences are labeled with different letters (*p* < 0.05) as result of a one-way ANOVA. OP, orthophosphate; PA, phytic acid; GOE-PA, phytic acid bound to goethite; GOE-OP, orthophosphate bound to goethite; GOE, mycorrhized plant, no P, and goethite; M+, mycorrhized plant, no P, quartz sand; M-, non-mycorrhized plant, no P, quartz sand.

**FIGURE 4:**
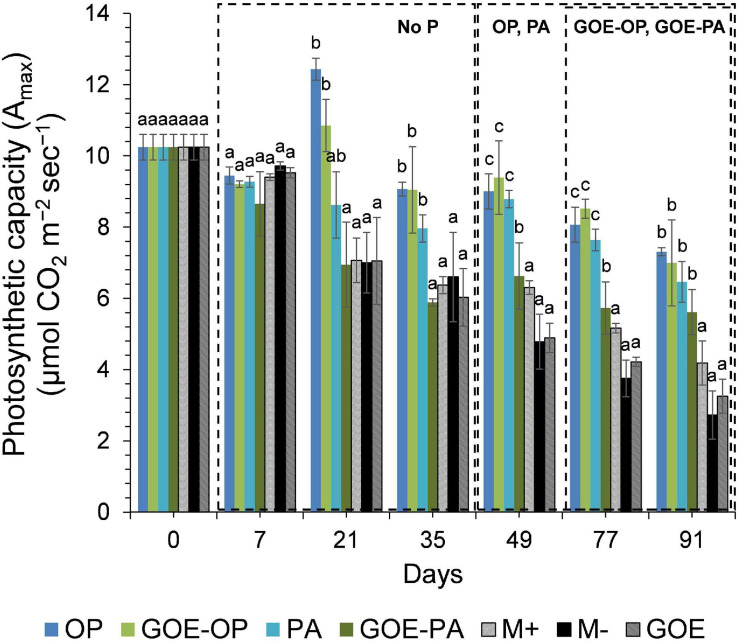
Mean values and standard errors for photosynthetic capacity (*A*_max_) of *Solanum lycopersicum* L. leaves for the different available P sources and controls during the time course experiment. Three explicative boxes are included to differentiate the periods when we detected the AM plants acquired P from their respective sources. Within each P source and day, treatments with significant differences are labeled with different letters (*p* < 0.05) as result of a one-way ANOVA. OP, orthophosphate; PA, phytic acid; GOE-PA, phytic acid bound to goethite; GOE-OP, orthophosphate bound to goethite; GOE, mycorrhized plant, no P, and goethite; M+, mycorrhized plant, no P, quartz sand; M-, non-mycorrhized plant, no P, quartz sand.

Mycorrhizal plants with access to free OP and PA exhibited P deficiency from day 21 until day 35, as was reflected by their N:P ratios >20 (Figure 3). From day 49 to day 91, AM plants with access to OP were not P-deficient, as indicated by mean N:P values <20. In the case of AM plants accessing the FC containing PA, the N:P ratios were stable from day 49 until day 91, showing a slight P deficiency in the plant tissues. The AM plants with access to GOE-OP and GOE-PA exhibited P deficiency from day 35 until day 49. At day 77, AM plants accessing both P forms bound to GOE showed a slight P deficiency with N:P values close to 20. From day 21 until day 91, all controls exhibited a significantly higher P deficiency, i.e., higher N:P ratios, compared to all treatments that accessed a P source (Figure 3). All controls with no access to a P source showed a significantly lower photosynthetic capacity (μmol CO_2_ m^–2^ s^–1^) from day 21 until day 91, compared to all treatments that accessed P sources (Figure 4).

### Low-Molecular-Weight Organic Acids in the FC

**FIGURE 5:**
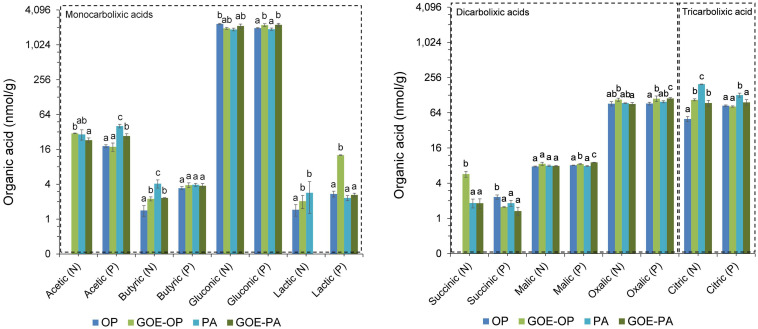
Low-molecular-weight organic acids (LMWOAs) determined in the fungal compartment containing the different available P sources during the time course experiment. LMWOA are grouped depending on the number of carboxylic groups (–COOH) present in their molecular structure, differentiating between mono/di/tricarboxylic acids. Based on the results from Figure 2, each bar shows the mean value and standard errors for all those samples which belong either to a period of no P incorporation (N) or to a period when we detected P incorporation (P), for their respective P sources. Within each test, treatments with significant differences are labeled with different letters (*p* < 0.05) as result of a one-way ANOVA. OP, orthophosphate; PA, phytic acid; GOE-PA, phytic acid bound to goethite; GOE-OP, orthophosphate bound to goethite.

Acetic and gluconic acids dominated the group of monocarboxylic acids, while oxalic and citric acids dominated the group of dicarboxylic and tricarboxylic acids, respectively (Figure 5). Mesocosms containing free OP in the FC only showed significantly larger contents of gluconic acid before P was allocated to the plant tissues. The treatment containing PA showed a significantly larger content of acetic, butyric, lactic, and citric acids before P uptake by the AM plant, while afterward only acetic and citric acids were present. The treatment containing GOE-OP exhibited a significantly larger content of all monocarboxylic acids, as well succinic, oxalic, and citric acids before P uptake by the AM plant. After the AM plants acquired P from GOE-OP, gluconic, lactic, malic, and oxalic acids were observed (Figure 5). Compared to the controls, the FC containing GOE-PA showed a significantly larger content of butyric, gluconic, and citric acids before the AM plant acquired P, while after P incorporation acetic, gluconic, lactic, malic, and oxalic acids were found in the FC.

### Fungal Growth and Degree of Mycorrhization

Of the four AMF PLFA markers (16:1ω5c, 18:1ω7c, 18:2ω6,9, 18:1ω9c), only the three first ones exhibited a significant positive correlation (*p* < 0.05) with the P incorporation in the plant tissues (Table 2). The AMF PLFA biomarker 16:1ω5c always correlated positively with acquired P in the whole plant for the four P forms (Table 2). Additionally, treatments with access to GOE-OP showed a significant correlation between the AMF PLFA biomarker 18:1ω7c with P incorporation, while the GOE-PA treatment did it with the AMF PLFA biomarker 18:2ω6,9.

**TABLE 2:** Result of the correlations between the fungal PLFA biomarkers and the acquired P (mg/plant).

**Fungal biomarker**	**Acquired P (mg/plant)**			
	**OP**	**PA**	**GOE-OP**	**GOE-PA**
16:1ω5c	0.662**	0.686**	0.737**	0.780**
18:2ω6,9	−0.581*	−0.4002	−0.1088	0.595*
18:1ω9c	0.103	−0.251	−0.482	−0.486
18:1ω7c	0.358	0.074	0.798**	−0.232

Correlation values in bold are significant (*p < 0.05, **p < 0.01). OP, orthophosphate; PA, phytic acid; GOE-PA, phytic acid bound to goethite; GOE-OP, orthophosphate bound to goethite.

In all tomato roots mycorrhized with *R. irregularis*, we observed an arum-type AM association (Saito and Ezawa, 2016) (Supplementary Table 1). Correlation analysis of AMF-derived PLFA and NLFA biomarkers with the P acquired into the plant tissues, and mycorrhizal root traits revealed that there was a significant positive correlation between the abundance of arbuscules (%) and the acquired P over time for all offered P sources. A similar positive correlation was found for the 16:1ω5c PLFA and the abundance of arbuscules (%). All mycorrhizal root traits of AM plants accessing GOE-PA and GOE-OP correlated positively with the NLFA 16:1ω5c. Plants mobilizing P sources bound to GOE also showed a significant positive correlation between mycorrhizal root traits and the PLFA and NLFA 18:1ω7c, 18:2ω6,9, respectively (Table 3). The fatty acid 18:1ω9c did not show any significant correlation with the P incorporation from any of the P sources (Table 3); thus, it was not included in the correlation analysis between mycorrhizal root status parameters and fungal biomarkers within the FC (Table 3).

**TABLE 3:** Result of the correlations between mycorrhization parameters and fungal biomarkers (%) at the fungal compartment.

		** PLFA (%)**			** NLFA (%)**			
		**Inc. P (mg)**	**16:1ω5c**	**18:2ω6,9**	**18:1ω7c**	**16:1ω5c**	**18:2ω6,9**	**18:1ω7c**
OP	A%	0.711**	0.565*	−0.326	0.621*	0.342	0.547*	0.469
	V%	0.444	0.116	−0.209	0.322	0.156	0.184	0.226
	M%	0.664**	0.430	−0.316	0.544*	0.279	0.424	0.412
PA	A%	0.771**	0.548*	−0.210	−0.072	0.414	0.121	0.380
	V%	0.429	0.396	0.052	−0.034	0.432	0.439	0.530*
	M%	0.533*	0.430	−0.016	0.027	0.441	0.357	0.522*
GOE-OP	A%	0.620**	0.587*	0.133	0.637*	0.938***	0.767**	0.932***
	V%	0.008	0.267	0.258	0.206	0.838***	0.479	0.779**
	M%	0.218	0.372	0.189	0.400	0.910***	0.599	0.868***
GOE-PA	A%	0.560*	0.774**	0.597*	−0.005	0.815**	0.483	0.801**
	V%	0.540*	0.691**	0.633*	−0.045	0.809**	0.439	0.820**
	M%	0.654*	0.743**	0.628*	−0.031	0.780**	0.478	0.787**

Correlation values in bold are significant (*p < 0.05, **p < 0.01, ***p < 0.001). PLFA, phospholipid fatty acid; NLFA, neutral lipid fatty acid; OP, orthophosphate; PA, phytic acid; GOE-PA, phytic acid bound to goethite; GOE-OP, orthophosphate bound to goethite; A, arbuscules; V, vesicles; M, mycorrhization; Inc. P, acquired P.

## Discussion

In the current study, we investigated the role of LMWOAs secreted by AMF and their accompanying microbiota in the mobilization of GOE-bound P sources. *R. irregularis* DAOM 197198 seemed not to be a specialist species in terms of mobilizing P bound to Fe oxides, since AM plants did not restore their initial P tissue contents during the time course experiment (Figure 2). It is not surprising, as it is a frequent dweller in agricultural contexts, thus not likely to be a functional specialist (Köhl et al., 2016). Our results show that before any P was acquired by the plant, there was a dilution in the P contents of shoots and roots for all treatments. Phosphorus was preferentially stored in the shoots, showing no P deficiency in case of AM plant mobilizing OP and PA, and only a slight P deficiency in case of those accessing GOE-OP and GOE-PA, as indicated by the N:P ratios (Figure 3). Furthermore, the AM plants with access to a P source exhibited significant higher photosynthetic activities until the end of the experiment, compared to the controls (Figure 4). Phosphorus is a key limiting nutrient and plays an important role in photosynthesis and the production of carbohydrates (Thuynsma et al., 2016). Plants may cycle P more efficiently at low soil P levels, by exhibiting a higher resorption efficiency (Dalling et al., 2016; Rychter et al., 2016). Hidaka and Kitayama (2013) proposed that in P-poor soils, plants tend to allocate P to the shoots, for keeping their productivity and growth and reducing the demand for P. Mycorrhizal benefit on the host plant is usually greater when plants are P limited (Hoeksema et al., 2010; Johnson et al., 2014) and this is particularly applicable to AM plants, which tend to store more P in the shoots, as compared to non-mycorrhizal plants (Yang et al., 2014; Holste et al., 2016). Furthermore, plants establishing AM symbiosis exhibit higher photosynthetic capacity, stomatal conductance, and transpiration rates, compared to non-mycorrhizal ones (Augé et al., 2016). The strength of the C sink in the mycorrhizal roots enhances plant photosynthetic capacity by wider opening of the stomata, allowing for more CO_2_ to diffuse into the leaf, which in terms increase the level of sucrose and hexose in roots (Boldt et al., 2011). Furthermore, the C sink to the roots accelerates the utilization of triose phosphate for sucrose synthesis and the export toward the phloem. This increases plant P recycling rates by releasing P back to the chloroplast and activating the regeneration of ribulose 1,5-bisphosphate in the Calvin cycle. By this mechanism, more C is fixed per time and per unit of P, resulting in higher photosynthetic P-use efficiency (Tuomi et al., 2001; Valentine et al., 2001; Kaschuk et al., 2009). Our results on the reduction of the P dilution in the shoots, coupled with lower N:P ratios and higher photosynthetic capacities over time, in those treatments with access to the GOE-bound P sources, may be an indicator that AM symbiosis was conducive to more efficient use of P mobilized from less-accessible sources. A more efficient photosynthetic P use may also benefit the secretion of LMWOAs, as they entail a substantial C cost which is exclusively supported by the direct supply of photoassimilates (Plassard and Fransson, 2009).

Studies on the exudation of LMWOA by AMF are somewhat limited, and we found none where these mediated the mobilization of P bound to iron oxides. Conversely, D’Amico et al. (2020) recently demonstrated that in extremely P-poor environments, ectomycorrhizal fungi were able to release both Pi and Po from associations with goethite. We found two similar studies where AMF growth was isolated and organic acid production was measured: The one by Toljander et al. (2007) detected the presence of acetate and formiate, and other by Tawaraya et al. (2006) observed citrate and malate as part of the AMF hyphal exudates. We also detected three out of the four LMWOAs found in the two previous studies in similar concentration as for citric acid (100 nmol/g FC substrate). Furthermore, we found couple of studies investigating P desorption from goethite with non-mycorrhizal plants and incubation experiments. Parfitt (1979) concluded that one of the mechanisms by which GOE-OP could be solubilized and made available is through the action of the LMWOAs. He performed several extraction cycles on GOE-OP in combination with different LMWOAs, resulting in a higher OP desorption when it was incubated in the presence of citrate. In a more recent study, Martin et al. (2004) studied the effects of LMWOAs which may be released by non-mycorrhizal plant roots, such as citrate, on the desorption of PA and OP bound to GOE. They found a smaller amount of PA desorbed from GOE as compared to OP, which was attributed to the strength of chemical bonds and the high negative charge of the complexes. In our experimental setup, we detected the presence of significantly larger concentrations of LMWOAs in the FCs containing PA, GOE-OP, and GOE-PA before and after any P was allocated to the plant tissues, compared to those ones containing OP (Figures 2, 5). The FCs where AM plants mobilized GOE-OP and GOE-PA contained the highest concentrations of malic and oxalic acid during the plant P incorporation phase. For citric acid, the trend was opposite to that of the dicarboxylic acids, having a significant larger concentration before any P was acquired in case of GOE-OP and GOE-PA treatments. The LMWOAs detected in the FCs of GOE-OP and GOE-PA treatments belong to the ones with higher chelation capacity, namely, malate, oxalate, and citrate, thus more effective mobilizing P from GOE or amorphous ferric hydroxides, as compared to the ones containing one carboxyl group (Muthukumar et al., 2014; Thorley et al., 2015). The release of mono/di/tri LMWOAs by the fungal partner of mycorrhizal plants refers to a possible mechanism involved in the acquisition of P from mineral-bound sources, where organic acids weaken and break the bonds between surface-coordinated P forms and structural metal ions before being mobilized by the AM plant. The presence of low contents of monocarboxylic acids (i.e., acetic, butyric, and lactic acids) before and after the AM plant acquired P from GOE-OP and GOE-PA, may be indicative of fermentation reactions occurring during transient periods of anaerobiosis, when reconstituting the water content to field capacity in the FCs. Although thermodynamically stable, goethite may undergo reductive dissolution in an anoxic environment when the redox potential drops (Torrent et al., 1987). This partial reductive dissolution may have taken place in FCs containing GOE-P compounds caused by the anaerobic respiration of microorganisms, transferring electrons from organic compounds to the Fe(III)-oxides (Peiffer and Wan, 2016). This reaction may have contributed, together with the action of di/tricarboxylic acids, in releasing adsorbed P from the goethite surfaces. However, the presence of the di/tri LMWOAs may suggest a mechanism used by AMF to desorb P from GOE surfaces. This desorption is done either by ligand exchange or by dissolution and subsequent desorption of P through the action of LMWOAs, such as oxalic, succinic, and citric acids. In the case of those plants accessing GOE-PA, the desorbed PA may be mineralized through the action of phosphatases secreted by the AMF (Tisserant et al., 2012).

We inoculated the tomato plants with *R. irregularis* DOAM 197198 grown under xenic conditions; thus, the inoculum carried the microorganisms naturally associated with its hyphae. In this sense, our results of LMWOAs production have to be examined under the possible joint influence of the AMF and its accompanying microbiota. In this regard, Battini et al. (2016) isolated microbiota from *Rhizophagus intraradices* and found plant growth-promoting activities such as phytate mineralization, siderophore production, Pi solubilization, and LMWOA production in several representatives of Gram-positive (e.g., *Streptomyces* spp., *Arthrobacter* spp., *Nocardiodes* spp., and *Bacillus* spp.) and Gram-negative bacterial groups (e.g., *Sinorhizobium* spp.). Furthermore, Lecomte et al. (2011) and Selvakumar et al. (2016) reported bacteria closely associated with the mycelium of *R. irregularis* involved in the P mobilization from phytic acid. More recently, P transfer from phytate via AMF with the assistance of phytate-mineralizing bacteria was confirmed by Hara and Saito (2016). They isolated bacteria from the hyphosphere of the cosmopolitan AMF *R. irregularis* DAOM197198 and found that *Claroideoglomus etunicatum* can mineralize phytic acid. Taktek et al. (2015, 2017) and Wang et al. (2016) also isolated bacteria closely attached to the hyphosphere of *R. irregularis* DAOM197198. They showed that exudates from *R. irregularis* hyphae supported the growth and activity of bacteria with high potential for LMWOA production and Po mineralization. A possible mechanism used by the AMF and its accompanying microbiota to desorb P from the surface of GOE would involve the release of exudates containing LMWOAs. Following this, the desorbed OP could be taken up directly by the AM hyphae, while the desorbed PA still had to be hydrolyzed by phosphatases. As we observed in our results, it is likely that these previous steps delayed the incorporation of P mobilized from GOE-PA and GOE-OP into the plant tissues, compared to the other treatments (Figure 2). Additionally, several authors (Otani and Ae, 1999; Hayes et al., 2000; George et al., 2005) have pointed out that some LMWOAs (e.g., citric acid) have a synergistic effect on the secreted phosphatases (e.g., acid phosphatase), by changing the chemical structure or molecular size of the extracted Po and making it more accessible to enzymatic action. Summarizing, we found profiles of LMWOAs differing with the accessibilities of the offered P sources. The LMWOAs with two and three carboxylic groups were more abundant in case of P sources with lower P accessibility, before any P was acquired into the plant tissues. Hence, our results would point to a plant–fungus synchronous functioning that would adapt over time to respond to P accessibility in the soil. In this way, the mycorrhizal symbiosis would favor a more efficient P utilization, by maintaining an adequate photosynthetic capacity to ensure the soil volume exploration, together with the secretion of the LMWOAs.

Our results on the presence of AMF lipid biomarkers, together with the P acquisition from the different sources, highlight the central role played by *R. irregularis* in mobilizing P into the AM plant. This statement is founded on the fact that lipid biomarkers in the FC increased along with P in the plant tissues (Table 2); besides, both parameters positively correlated with the presence of arbuscules (Table 3). The arbuscules are short-lived structures with a turnover rate of 1–2 weeks (van Aarle and Olsson, 2003) and the interface between the plant and AMF (Wewer et al., 2014), where the P and photosynthates are exchanged in the periarbuscular space (Kobae et al., 2014; Saito and Ezawa, 2016). Thus, it would be consistent with the interpretation that P mobilization stimulated by the LMWOAs secretion was further supported by fungal growth and the exchange structures at the root level. The second conclusion stems from the correlation between the fungal PLFAs 18:1ω7c and 18:2ω6,9 with the acquired P and the arbuscules (%), for those AM plants mobilizing P from GOE-OP and GOE-PA, respectively. The PLFA are vital components of all biological membranes and play a key role in processes such a signal transduction, cytoskeletal rearrangement, membrane trafficking, etc., and remain at the place where they are synthesized (van Aarle and Olsson, 2003; Debiane et al., 2011; Dalpé et al., 2012). The AMF lack genes for a *de novo* biosynthesis of lipids and are enzymatically only able to elongate 16C lipid molecules; they mandatorily receive from their host plant (Bravo et al., 2017; Keymer et al., 2017). Since, AMF only elongates 16C lipid molecules, requiring the plant to produce them (Luginbuehl et al., 2017), the presence of the two unsaturated 18C PLFA fungal biomarkers in our experiment (18:1ω7c and 18:2ω6,9) might support the possibility of a modified composition in lipids constituting the hyphal membrane, which might be seen as an adaptation to the accessibility of the different P sources. Plasticity in fatty acid synthesis attributable to nutritional factors is common in filamentous fungi (Olsson et al., 2002). Based on the correlation data, our results point toward a change in the unsaturation level of AMF membrane lipids with the changing quality of the offered P sources. *R. irregularis*, therefore, might have modified its lipid composition in response to the different P sources. Consequently, the lipid membrane increased its fluidity to keep its integrity compatible with an optimal membrane functionality (Calonne et al., 2010). Membrane fluidity depends on its phospholipid composition of varying length and saturation with unsaturated lipid chains being more fluid than saturated ones. The unsaturated double bonds make it harder for the lipids to pack together by putting kinks into the otherwise straight hydrocarbon chain (Reichle, 1989). For successful adaptation to altered physicochemical environments, the active remodeling of membrane lipid composition is an essential feature and depends on both strain properties and cultivation conditions (Bentivenga and Morton, 1994; Čertík et al., 2005). Changes in membrane fluidity influence membrane processes such as transport, enzyme activities, and signal transduction (Benyagoub et al., 1996; Turk et al., 2007). In summary, it is plausible to consider that AMF modified its membrane lipid composition when mobilizing the GOE-bound P sources may have modulated the way in which the lipid membrane was organized for maintaining the growth state (Wang et al., 2017), by increasing unsaturated lipids in the case the AMF developed on a P source bound to GOE.

## Conclusion

We found that free P sources were earlier acquired by the AM plant compared to their goethite-associated counterparts. Our results on the acquisition of P from GOE-bound sources suggest the AM symbiosis was conducive to greater P-use efficiency. Since we found evidence pointing to a synchronous response of the plant–fungus binomial, by mobilizing P desorbed from GOE to the photosynthetically active tissues and ensuring an adequate photosynthetic capacity for fueling the exploration of hyphae in the soil, as well as the costly production of LMWOAs. The LMWOAs with two and three carboxylic groups (e.g., oxalic, succinic, and citric acids) were more abundant in those FCs where P was mobilized from sources with lower accessibility. This fact suggests that desorption of OP and PA from GOE was mediated either by ligand exchange or by ligand-controlled dissolution. Additionally, the presence of low contents of monocarboxylic acids characteristic of transient anaerobic conditions (i.e., acetic, butyric and lactic acids) before and after the AM plant acquired P from GOE-P associations may be indicative of reductive dissolution processes to release P from goethite surfaces. Finally, the fungal lipid analysis may indicate the AMF modified its membrane lipid composition by increasing the amount of unsaturated lipids when mobilizing the GOE-bound P sources, for maintaining the growth state and functionality. The AM symbiosis with *R. irregularis* and accompanying microbiota played a central role mobilizing P from GOE-bound sources to the host plant, highlighting the potentially pervasive influence of AMF on key ecosystem processes as the cycling of essential plant nutrients.

## Data Availability Statement

Raw and derived data supporting the findings of this study are available from the corresponding author AA on request.

## Author Contributions

AA, JB, GG, RM, and LS designed the experiment. AA prepared the plant and fungal material, conducted the experiment, analyzed the data, and wrote the manuscript with contributions from JB, GG, RM, LS, and SK. SK analyzed the LMWOAs on the FC samples and described the material and methods. JB, GG, LS, and RM supervised the research. All authors contributed to the article and approved the submitted version.

## Conflict of Interest

The authors declare that the research was conducted in the absence of any commercial or financial relationships that could be construed as a potential conflict of interest.

## References

[B1] Adeleke R. Nwangburuka C. Oboirien B. (2017). Origins, roles and fate of organic acids in soils: a review. *S. Afric. J. Bot.* 108 393–406. 10.1016/j.sajb.2016.09.002

[B2] Andrino A. Boy J. Mikutta R. Sauheitl L. Guggenberger G. (2019). Carbon investment required for the mobilization of inorganic and organic phosphorus bound to goethite by an arbuscular mycorrhiza (*Solanum lycopersicum* x *Rhizophagus irregularis*). *Front. Environ. Sci.* 7:26. 10.3389/fenvs.2019.00026

[B3] Andrino A. Guggenberger G. Sauheitl L. Burkart S. Boy J. (2020). Carbon investment into mobilization of mineral and organic phosphorus by arbuscular mycorrhiza. *Biol. Fertil. Soils* 57 47–64. 10.1007/s00374-020-01505-5

[B4] Augé R. M. Toler H. D. Saxton A. M. (2016). Mycorrhizal stimulation of leaf gas exchange in relation to root colonization, shoot size, leaf phosphorus and nitrogen: a quantitative analysis of the literature using meta-regression. *Front. Plant Sci.* 7:1084. 10.3389/fpls.2016.01084 27524989 PMC4965464

[B5] Bååth E. (2003). The use of neutral lipid fatty acids to indicate the physiological conditions of soil fungi. *Microb. Ecol.* 45 373–383. 10.1007/s00248-003-2002-y 12704558

[B6] Battini F. Cristani C. Giovannetti M. Agnolucci M. (2016). Multifunctionality and diversity of culturable bacterial communities strictly associated with spores of the plant beneficial symbiont *Rhizophagus intraradices*. *Microbiol. Res.* 183 68–79. 10.1016/j.micres.2015.11.012 26805620

[B7] Bentivenga S. P. Morton J. B. (1994). Stability and heritability of fatty-acid methyl-ester profiles of Glomalean Endomycorrhizal fungi. *Mycol. Res.* 98 1419–1426. 10.1016/S0953-7562(09)81073-3

[B8] Benyagoub M. Willemot C. Bélanger R. R. (1996). Influence of a subinhibitory dose of antifungal atty acids from *Sporothrix flocculosa* on cellular lipid composition in fungi. *Lipids* 31 1077–1082. 10.1007/BF02522465 8898307

[B9] Bharadwaj D. P. Alström S. Lundquist P.-O. O. (2012). Interactions among *Glomus irregulare*, arbuscular mycorrhizal spore-associated bacteria, and plant pathogens under in vitro conditions. *Mycorrhiza* 22 437–447. 10.1007/s00572-011-0418-7 22081167

[B10] Bischoff N. Mikutta R. Shibistova O. Puzanov A. Reichert E. Silanteva M. (2016). Land-use change under different climatic conditions: consequences for organic matter and microbial communities in Siberian steppe soils. *Agric. Ecosyst. Environ.* 235 253–264. 10.1016/j.agee.2016.10.022

[B11] Boldt K. Pörs Y. Haupt B. Bitterlich M. Kühn C. Grimm B. (2011). Photochemical processes, carbon assimilation and RNA accumulation of sucrose transporter genes in tomato arbuscular mycorrhiza. *J. Plant Physiol.* 168 1256–1263. 10.1016/j.jplph.2011.01.026 21489650

[B12] Bravo A. Brands M. Wewer V. Dörmann P. Harrison M. J. (2017). Arbuscular mycorrhiza-specific enzymes FatM and RAM2 fine-tune lipid biosynthesis to promote development of arbuscular mycorrhiza. *New Phytol.* 214 1631–1645. 10.1111/nph.14533 28380681

[B13] Brundrett M. Bougher N. Dell B. Grove T. Malajczuk N. (1996). *Working with Mycorrhizas in Forestry and Agriculture.* Canberra: Australian Centre for International Agricultural Research.

[B14] Burghelea C. I. Dontsova K. Zaharescu D. G. Maier R. M. Huxman T. Amistadi M. K. (2018). Trace element mobilization during incipient bioweathering of four rock types. *Geochim. Cosmochim. Acta* 234 98–114. 10.1016/j.gca.2018.05.011

[B15] Calonne M. Fontaine J. J. Debiane D. Laruelle F. F. Grandmougin-ferjani A. Sahraoui A. L. (2010). “Propiconazole toxicity on the non-target organism, the arbuscular mycorrhizal fungus, glomus irregulare,” in *Fungicides*, ed. Carisse O. (London: InTech Open), 325–346.

[B16] Čertík M. Breierová E. Juršíková P. (2005). Effect of cadmium on lipid composition of *Aureobasidium pullulans* grown with added extracellular polysaccharides. *Int. Biodeterior. Biodegrad.* 55 195–202. 10.1016/j.ibiod.2004.11.005

[B17] Dalling J. Heineman K. Lopez O. Wright S. Turner B. (2016). “Nutrient availability in tropical rain forests: the paradigm of phosphorus limitation,” in *Tropical Tree Physiology*, eds Goldstein G. Santiago L. S. (Cham: Springer International Publishing), 261–273. 10.1007/978-3-319-27422-5_12

[B18] Dalpé Y. Trépanier M. Sahraoui A. L.-H. Fontaine J. Sancholle M. (2012). Lipids of Mycorrhizas. *Fungal Assoc.* 9 137–169. 10.1007/978-3-642-30826-0_8

[B19] D’Amico M. Almeida J. P. Barbieri S. Castelli F. Sgura E. Sineo G. (2020). Ectomycorrhizal utilization of different phosphorus sources in a glacier forefront in the Italian Alps. *Plant Soil* 446 81–95. 10.1007/s11104-019-04342-0

[B20] Debiane D. Calonne M. Fontaine J. Laruelle F. Grandmougin-Ferjani A. Lounes-Hadj Sahraoui A. (2011). Lipid content disturbance in the arbuscular mycorrhizal, *Glomus irregulare* grown in monoxenic conditions under PAHs pollution. *Fungal Biol.* 115 782–792. 10.1016/j.funbio.2011.06.003 21802059

[B21] Deubel A. Merbach W. (2005). “Influence of microorganisms on phosphorus bioavailability in soils,” in *Microorganisms in Soils: Roles in Genesis and Functions*, eds Varma A. Buscot F. (Berlin: Springer-Verlag), 177–191. 10.1007/3-540-26609-7_9

[B22] Drigo B. Donn S. (2017). “Trading carbon between arbuscular mycorrhizal fungi and their hyphae-associated microbes,” in *Mycorrhizal Mediation of Soil: Fertility, Structure, and Carbon Storage*, eds Johnson N. C. Gehring C. A. Jansa J. (Amsterdam: Elsevier), 395–412. 10.1016/B978-0-12-804312-7.00022-X

[B23] Fernández I. Merlos M. López-Ráez J. A. Martínez-Medina A. Ferrol N. Azcón C. (2014). Defense related phytohormones regulation in arbuscular mycorrhizal symbioses depends on the partner genotypes. *J. Chem. Ecol.* 40 791–803. 10.1007/s10886-014-0473-6 24997625

[B24] Fitter A. H. Graves J. D. Watkins N. K. Robinson D. Scrimgeour C. (1998). Carbon transfer between plants and its control in networks of arbuscular mycorrhizas. *Funct. Ecol.* 12 406–412. 10.1046/j.1365-2435.1998.00206.x

[B25] Frostegård Å Tunlid A. Bååth E. (1991). Microbial biomass measured as total lipid phosphate in soils of different organic content. *J. Microbiol. Methods* 14 151–163. 10.1016/0167-7012(91)90018-L

[B26] Geelhoed J. S. Van Riemsdijk W. H. Findenegg G. R. (1999). Simulation of the effect of citrate exudation from roots on the plant availability of phosphate adsorbed on goethite. *Eur. J. Soil Sci.* 50 379–390. 10.1046/j.1365-2389.1999.00251.x

[B27] George T. S. Simpson R. J. Hadobas P. A. Richardson A. E. (2005). Expression of a fungal phytase gene in *Nicotiana tabacum* improves phosphorus nutrition of plants grown in amended soils. *Plant Biotechnol. J.* 3 129–140. 10.1111/j.1467-7652.2004.00116.x 17168905

[B28] Gogala N. Virant-Klun I. Gogala N. (1995). Impact of vam on phosphorus nutrition of maize with low soluble phosphate fertilization. *J. Plant Nutr.* 18 1815–1823. 10.1080/01904169509365025

[B29] Güsewell S. (2004). N: P ratios in terrestrial plants: variation and functional significance. *New Phytol.* 164 243–266. 33873556 10.1111/j.1469-8137.2004.01192.x

[B30] Hara S. Saito M. (2016). Isolation of inositol hexaphosphate (IHP)-degrading bacteria from arbuscular mycorrhizal fungal hyphal compartments using a modified baiting method involving alginate beads containing IHP. *Microbes Environ.* 31 234–243. 10.1264/jsme2.ME15206 27383681 PMC5017799

[B31] Hayes J. E. E. Richardson A. E. E. Simpson R. J. J. (2000). Components of organic phosphorus in soil extracts that are hydrolysed by phytase and acid phosphatase. *Biol. Fertil. Soils* 32 279–286. 10.1007/s003740000249

[B32] Hayes P. Turner B. L. Lambers H. Laliberté E. (2014). Foliar nutrient concentrations and resorption efficiency in plants of contrasting nutrient-acquisition strategies along a 2-million-year dune chronosequence. *J. Ecol.* 102 396–410. 10.1111/1365-2745.12196

[B33] He Z. Zhu J. U. N. (1998). Microbial utilization and transformation of phosphate adsorbed by variable charge minerals. *Soil Biol. Biochem.* 30 917–923. 10.1016/S0038-0717(97)00188-0

[B34] Herrera-Medina M. J. Tamayo M. I. Vierheilig H. Ocampo J. A. García-Garrido J. M. (2008). The jasmonic acid signalling pathway restricts the development of the arbuscular mycorrhizal association in tomato. *J. Plant Growth Regul.* 27 221–230. 10.1007/s00344-008-9049-4

[B35] Hewitt E. J. (1966). Sand and water culture methods used in the study of plant nutrition. *Soil Sci. Soc. Am.* 17 301–301.

[B36] Hidaka A. Kitayama K. (2013). Relationship between photosynthetic phosphorus-use efficiency and foliar phosphorus fractions in tropical tree species. *Ecol. Evol.* 3 4872–4880. 10.1002/ece3.861 24455122 PMC3892354

[B37] Hoeksema J. D. Chaudhary V. B. Gehring C. A. Johnson N. C. Karst J. Koide R. T. (2010). A meta-analysis of context-dependency in plant response to inoculation with mycorrhizal fungi. *Ecol. Lett.* 13 394–407. 10.1111/j.1461-0248.2009.01430.x 20100237

[B38] Holste E. K. Kobe R. K. Gehring C. A. (2016). Plant species differ in early seedling growth and tissue nutrient responses to arbuscular and ectomycorrhizal fungi. *Mycorrhiza* 27 211–223. 10.1007/s00572-016-0744-x 27838856

[B39] IBM Corporation (2016). *IBM SPSS Statistics for Windows, Version 24.0, 2016.* Armonk, NJ: IBM Corporation.

[B40] Johansen A. Finlay R. D. Olsson P. A. (1996). Nitrogen metabolism of external hyphae of the arbuscular mycorrhizal fungus *Glomus intraradices*. *New Phytol.* 133 705–712. 10.1111/j.1469-8137.1996.tb01939.x

[B41] Johnson N. C. Wilson G. W. T. Wilson J. A. Miller R. M. Bowker M. A. (2014). Mycorrhizal phenotypes and the law of the minimum. *New Phytol.* 205 1473–1484. 10.1111/nph.13172 25417818

[B42] Kaschuk G. Kuyper T. W. Leffelaar P. A. Hungria M. Giller K. E. (2009). Are the rates of photosynthesis stimulated by the carbon sink strength of Rhizobial and arbuscular mycorrhizal symbioses? *Soil Biol. Biochem.* 41 1233–1244. 10.1016/j.soilbio.2009.03.005

[B43] Keymer A. Pimprikar P. Wewer V. Huber C. Brands M. Bucerius S. L. (2017). Lipid transfer from plants to arbuscular mycorrhiza fungi. *eLife* 6:e29107. 10.7554/eLife.29107 28726631 PMC5559270

[B44] Klugh K. R. Cumming J. R. (2007). Variations in organic acid exudation and aluminum resistance among arbuscular *Mycorrhizal* species colonizing *Liriodendron tulipifera*. *Tree Physiol.* 27 1103–1112. 10.1093/treephys/27.8.1103 17472937

[B45] Kobae Y. Gutjahr C. Paszkowski U. Kojima T. Fujiwara T. Hata S. (2014). Lipid droplets of arbuscular mycorrhizal fungi emerge in concert with arbuscule collapse. *Plant Cell Physiol.* 55 1945–1953. 10.1093/pcp/pcu123 25231957

[B46] Köhl L. Lukasiewicz C. E. Van der Heijden M. G. A. (2016). Establishment and effectiveness of inoculated arbuscular mycorrhizal fungi in agricultural soils. *Plant Cell Environ.* 39 136–146. 10.1111/pce.12600 26147222

[B47] Lecomte J. St-Arnaud M. Hijri M. (2011). Isolation and identification of soil bacteria growing at the expense of arbuscular mycorrhizal fungi. *FEMS Microbiol. Lett.* 317 43–51. 10.1111/j.1574-6968.2011.02209.x 21219415

[B48] Luginbuehl L. H. Menard G. N. Kurup S. Van Erp H. Radhakrishnan G. V. Breakspear A. (2017). Fatty acids in arbuscular mycorrhizal fungi are synthesized by the host plant. *Science* 356 1175–1178. 10.1126/science.aan0081 28596311

[B49] Mäder P. Vierheilig H. Streitwolf-Engel R. Boller T. Frey B. Christie P. (2000). Transport of 15N from a soil compartment separated by a polytetrafluoroethylene membrane to plant roots via the hyphae of arbuscular mycorrhizal fungi. *New Phytol.* 146 155–161. 10.1046/j.1469-8137.2000.00615.x

[B50] Manchanda G. Singh R. P. Li Z. F. Zhang J. J. (2017). “Mycorrhiza: creating good spaces for interactions,” in *Mycorrhiza - Function, Diversity, State of the Art*, eds Varma A. Prasad R. Tuteja N. (Cham: Springer International Publishing), 39–60. 10.1007/978-3-319-53064-2_4

[B51] Marschener H. (1998). Role of root growth, arbuscular mycorrhiza, and root exudates for the efficiency in nutrient acquisition. *Field Crop. Res.* 56 203–207. 10.1016/S0378-4290(97)00131-7

[B52] Martin M. Celi L. Barberis E. (2004). Desorption and plant availability of myo-inositol hexaphosphate adsorbed on goethite. *Soil Sci.* 169 115–124. 10.1097/01.ss.0000117787.98510.9d

[B53] McCormack M. L. Iversen C. M. (2019). Physical and functional constraints on viable belowground acquisition strategies. *Front. Plant Sci.* 10:1215. 10.3389/fpls.2019.01215 31681355 PMC6797606

[B54] McGonigle T. P. Miller M. H. Evans D. G. Fairchild G. L. Swan J. A. (1990). A new method which gives an objective measure of colonization of roots by vesicular—arbuscular mycorrhizal fungi. *New Phytol.* 115 495–501. 10.1111/j.1469-8137.1990.tb00476.x 33874272

[B55] Muthukumar T. Priyadharsini P. Uma E. Jaison S. Pandey R. R. (2014). “Role of arbuscular mycorrhizal fungi in alleviation of acidity stress on plant growth,” in *Use of Microbes for the Alleviation of Soil Stresses*, Vol. 1 ed. Miransari M. (New York, NY: Springer), 43–71. 10.1007/978-1-4614-9466-9_3

[B56] Oberson A. Friesen D. K. Rao I. M. M. Bühler S. Frossard E. Buhler S. (2001). “Phosphorus transformations in an oxisol under contrasting land-use systems: the role of the soil microbial biomass,” in *Plant and Soil*, (Amsterdam: Kluwer Academic Publishers), 197–210. 10.1023/A:1013301716913

[B57] Oburger E. Jones D. L. Wenzel W. W. (2011). Phosphorus saturation and pH differentially regulate the efficiency of organic acid anion-mediated P solubilization mechanisms in soil. *Plant Soil* 341 363–382. 10.1007/s11104-010-0650-5

[B58] Olsson P. A. Johansen A. (2000). Lipid and fatty acid composition of hyphae and spores of arbuscular mycorrhizal fungi at different growth stages. *Mycol. Res.* 104 429–434. 10.1017/S0953756299001410

[B59] Olsson P. A. van Aarle I. M. Allaway W. G. Ashford A. E. A. E. Rouhier H. (2002). Phosphorus effects on metabolic processes in Monoxenic Arbuscular Mycorrhiza cultures. *Plant Physiol.* 130 1162–1171. 10.1104/pp.009639 12427983 PMC166637

[B60] Olsson P. A. Wilhelmsson P. (2000). The growth of external AM fungal mycelium in sand dunes and in experimental systems. *Plant Soil* 226 161–169. 10.1023/A:1026565314345

[B61] Otani T. Ae N. (1999). Extraction of organic phosphorus in Andosols by various methods. *Soil Sci. Plant Nutr.* 45 151–161. 10.1080/00380768.1999.10409331

[B62] Owen D. Williams A. P. Griffith G. W. Withers P. J. A. (2015). Use of commercial bio-inoculants to increase agricultural production through improved phosphrous acquisition. *Appl. Soil Ecol.* 86 41–54. 10.1016/j.apsoil.2014.09.012

[B63] Parfitt R. L. (1979). The availability of P from phosphate-goethite bridging complexes. Desorption and uptake by ryegrass. *Plant Soil* 53 55–65. 10.1007/BF02181879

[B64] Peiffer S. Wan M. (2016). “Reductive dissolution and reactivity of Ferric (Hydr)Oxides: new insights and implications for environmental Redox processes,” in *Iron Oxides: From Nature to Applications*, ed. Faivrein D. (Hoboken, NJ: Wiley), 31–52. 10.1002/9783527691395.ch3

[B65] Plassard C. Fransson P. (2009). Regulation of low-molecular weight organic acid production in fungi. *Fungal Biol. Rev.* 23 30–39. 10.1016/j.fbr.2009.08.002

[B66] Rakshit A. Bhadoria P. S. (2010). Role of VAM on growth and phosphorus nutrition of maize with low soluble phosphate fertilization. *Acta Agronóm.* 59 119–123.

[B67] Reichle D. E. (1989). “Biomembranes: molecular structure and function,” in *Springer Advanced Texts in Chemistry*, (Berkeley, CA: University of California Press), 533. 10.1007/978-1-4757-2065-5

[B68] Richardson A. E. (2001). Prospects for using soil microorganisms to improve the acquisition of phosphorus by plants. *Aust. J. Plant Physiol.* 28 897–906. 10.1071/PP01093

[B69] Ros M. B. H. De Deyn G. B. Koopmans G. F. Oenema O. van Groenigen J. W. (2018). What root traits determine grass resistance to phosphorus deficiency in production grassland? *J. Plant Nutr. Soil Sci.* 181 323–335. 10.1002/jpln.201700093

[B70] Rychter A. Rao I. Cardoso J. (2016). “Role of phosphorus in photosynthetic carbon assimilation and partitioning,” in *Handbook of Photosynthesis*, ed. Pessarakli M. (Boca Raton, FL: CRC Press), 603–625. 10.1201/b19498-45

[B71] Saito K. Ezawa T. (2016). “Phosphorus metabolism and transport in arbuscular mycorrhizal symbiosis,” in *Molecular Mycorrhizal Symbiosis*, (Hoboken, NJ: John Wiley & Sons, Inc.), 197–216. 10.1002/9781118951446.ch12

[B72] Sato T. Ezawa T. Cheng W. Tawaraya K. (2015). Release of acid phosphatase from extraradical hyphae of arbuscular mycorrhizal fungus *Rhizophagus clarus*. *Soil Sci. Plant Nutr.* 61 269–274. 10.1080/00380768.2014.993298 31745622

[B73] Schachtman D. P. Reid R. J. Ayling S. M. (1998). Update on phosphorus uptake phosphorus uptake by plants: from soil to cell. *Plant Physiology* 116 447–453. 9490752 10.1104/pp.116.2.447 PMC1539172

[B74] Seguel A. Cumming J. R. Klugh-Stewart K. Cornejo P. Borie F. (2013). The role of arbuscular mycorrhizas in decreasing aluminium phytotoxicity in acidic soils: a review. *Mycorrhiza* 23 167–183. 10.1007/s00572-013-0479-x 23328806

[B75] Selvakumar G. Krishnamoorthy R. Kim K. Sa T. M. (2016). Genetic diversity and association characters of bacteria isolated from arbuscular mycorrhizal fungal spore walls. *PLoS One* 11:e0160356. 10.1371/journal.pone.0160356 27479250 PMC4968797

[B76] Smith S. Read D. (2008). *Mycorrhizal Symbiosis.* New York, NY: Academic Press. 10.1016/B978-0-12-370526-6.X5001-6

[B77] Taktek S. St-Arnaud M. Piché Y. Fortin J. A. Antoun H. (2017). Igneous phosphate rock solubilization by biofilm-forming mycorrhizobacteria and hyphobacteria associated with *Rhizoglomus irregulare* DAOM 197198. *Mycorrhiza* 27 13–22. 10.1007/s00572-016-0726-z 27541158 PMC5203815

[B78] Taktek S. Trépanier M. Servin P. M. St-Arnaud M. Piché Y. Fortin J.-A. A. (2015). Trapping of phosphate solubilizing bacteria on hyphae of the arbuscular mycorrhizal fungus *Rhizophagus irregularis* DAOM 197198. *Soil Biol. Biochem.* 90 1–9. 10.1016/j.soilbio.2015.07.016

[B79] Tani M. Higashi T. (1999). Vertical distribution of low molecular weight aliphatic carboxylic acids in some forest soils of Japan. *Eur. J. Soil Sci.* 50 217–226. 10.1046/j.1365-2389.1999.00228.x

[B80] Tawaraya K. Naito M. Wagatsuma T. (2006). Solubilization of insoluble inorganic phosphate by hyphal exudates of arbuscular mycorrhizal fungi. *J. Plant Nutr.* 29 657–665. 10.1080/01904160600564428

[B81] Thorley R. M. S. Taylor L. L. Banwart S. A. Leake J. R. Beerling D. J. (2015). The role of forest trees and their mycorrhizal fungi in carbonate rock weathering and its significance for global carbon cycling. *Plant. Cell Environ.* 38 1947–1961. 10.1111/pce.12444 25211602

[B82] Thuynsma R. Kleinert A. Kossmann J. Valentine A. J. Hills P. N. (2016). The effects of limiting phosphate on photosynthesis and growth of *Lotus japonicus*. *S. Afric. J. Bot.* 104 244–248. 10.1016/j.sajb.2016.03.001

[B83] Tisserant E. Kohler A. Dozolme-Seddas P. Balestrini R. Benabdellah K. Colard A. (2012). The transcriptome of the arbuscular mycorrhizal fungus *Glomus intraradices* (DAOM 197198) reveals functional tradeoffs in an obligate symbiont. *New Phytol.* 193 755–769. 10.1111/j.1469-8137.2011.03948.x 22092242

[B84] Toljander J. F. Lindahl B. D. Paul L. R. Elfstrand M. Finlay R. D. (2007). Influence of arbuscular mycorrhizal mycelial exudates on soil bacterial growth and community structure. *FEMS Microbiol. Ecol.* 61 295–304. 10.1111/j.1574-6941.2007.00337.x 17535297

[B85] Torrent J. Schwertmann U. Barron V. (1987). The reductive dissolution of synthetic goethite and hematite in dithionite. *Clay Miner.* 22 329–337. 10.1180/claymin.1987.022.3.07

[B86] Tuomi J. Kytoviita M.-M. M. Hardling R. (2001). Cost efficiency of nutrient acquisition and the advantage of mycorrhizal symbiosis for the host plant. *Oikos* 92 62–70. 10.1034/j.1600-0706.2001.920108.x 11841302

[B87] Turk M. Abramović Z. Plemenitaš A. Gunde-Cimerman N. (2007). Salt stress and plasma-membrane fluidity in selected extremophilic yeasts and yeast-like fungi. *FEMS Yeast Res.* 7 550–557. 10.1111/j.1567-1364.2007.00209.x 17298474

[B88] Valentine A. J. Osborne B. A. Mitchell D. T. (2001). Interactions between phosphorus supply and total nutrient availability on mycorrhizal colonization, growth and photosynthesis of cucumber. *Sci. Hortic.* 88 177–189. 10.1016/S0304-4238(00)00205-3

[B89] van Aarle I. M. Olsson P. A. (2003). Fungal lipid accumulation and development of mycelial structures by two arbuscular mycorrhizal. *Appl. Environ. Microbiol.* 69 6762–6767. 10.1128/AEM.69.11.6762 14602638 PMC262256

[B90] Vierheilig H. Coughlan A. P. Wyss U. Piché Y. (1998). Ink and vinegar, a simple staining technique for arbuscular-mycorrhizal fungi. *Appl. Environ. Microbiol.* 64 5004–5007. 9835596 10.1128/aem.64.12.5004-5007.1998 PMC90956

[B91] Wang F. Shi N. Jiang R. Zhang F. Feng G. (2016). In situ stable isotope probing of phosphate-solubilizing bacteria in the hyphosphere. *J. Exp. Bot.* 67 1689–1701. 10.1093/jxb/erv561 26802172 PMC4783358

[B92] Wang J. Chen W. Nian H. Ji X. Lin L. Wei Y. (2017). Inhibition of polyunsaturated fatty acids synthesis decreases growth rate and membrane fluidity of *Rhodosporidium kratochvilovae* at low temperature. *Lipids* 52 729–735. 10.1007/s11745-017-4273-y 28660529

[B93] Wang P. Wang T. Y. Wu S. H. Wen M. X. Lu L. M. Ke F. Z. (2019). Effect of arbuscular mycorrhizal fungi on rhizosphere organic acid content and microbial activity of trifoliate orange under different low P conditions. *Arch. Agron. Soil Sci.* 65 2029–2042. 10.1080/03650340.2019.1590555

[B94] Watkins N. K. Fitter A. H. Graves J. D. Robinson D. (1996). Carbon transfer between C3 and C4 plants linked by a common mycorrhizal network, quantified using stable carbon isotopes. *Soil Biol. Biochem.* 28 471–477. 10.1016/0038-0717(95)00189-1

[B95] Wewer V. Brands M. Dörmann P. (2014). Fatty acid synthesis and lipid metabolism in the obligate biotrophic fungus *Rhizophagus irregularis* during mycorrhization of *Lotus japonicus*. *Plant J.* 79 398–412. 10.1111/tpj.12566 24888347

[B96] Xu G. H. Chague V. Melamed-Bessudo C. Kapulnik Y. Jain A. Raghothama K. G. (2007). Functional characterization of LePT4: a phosphate transporter in tomato with mycorrhiza-enhanced expression. *J. Exp. Bot.* 58 2491–2501. 10.1093/jxb/erm096 17545228

[B97] Yang Y. Tang M. Sulpice R. Chen H. Tian S. Ban Y. (2014). Arbuscular Mycorrhizal fungi alter fractal dimension characteristics of *Robinia pseudoacacia* L. seedlings through regulating plant growth, leaf water status, photosynthesis, and nutrient concentration under drought stress. *J. Plant Growth Regul.* 33 612–625. 10.1007/s00344-013-9410-0

[B98] Zhang L. Fan J. Ding X. He X. Zhang F. Feng G. (2014). Hyphosphere interactions between an arbuscular mycorrhizal fungus and a phosphate solubilizing bacterium promote phytate mineralization in soil. *Soil Biol. Biochem.* 74 177–183. 10.1016/j.soilbio.2014.03.004

[B99] Zhang L. Xu M. Liu Y. Zhang F. Hodge A. Feng G. (2016). Carbon and phosphorus exchange may enable cooperation between an arbuscular mycorrhizal fungus and a phosphate-solubilizing bacterium. *New Phytol.* 210 1022–1032. 10.1111/nph.13838 27074400

