# Supplementary material for: Production of Organic Acids by Arbuscular Mycorrhizal Fungi and Their Contribution in the Mobilization of Phosphorus Bound to Iron Oxides
Source: Front Plant Sci. 2021 Jul 15;12:661842. doi: 10.3389/fpls.2021.661842 (PMC8320662; doi:10.3389/fpls.2021.661842)
Supplement: Supplementary file 1 [file Data_Sheet_1.docx]

**Supplementary materials**

**Production of organic acids by arbuscular mycorrhizal fungi and their contribution in the mobilization of phosphorus bound to iron oxides**

Alberto Andrino^1^, Georg Guggenberger^1^, Sarmite Kernchen^2^, Robert Mikutta^3^, Leopold Sauheitl^1^ and Jens Boy^1^

^1^Institute of Soil Science, Leibniz Universität Hannover, Herrenhäuser Straße 2, 30419 Hannover, Germany.

^2^Bayreuth Center of Ecology and Environmental Research (BayCEER), Bayreuth University, Dr. Hans Frisch Straße B3, 95448 Bayreuth, Germany.

^3^Soil Science and Soil Protection, Martin Luther University Halle-Wittenberg, von-Seckendorff-Platz 3, 06210 Halle (Saale), Germany.

**Corresponding author:** andrino@ifbk.uni-hannover.de

**Keywords**

Arbuscular mycorrhiza, organic acid, organic phosphorus (Po), inorganic phosphorus (Pi), iron oxides, reductive dissolution, ligand exchange, membrane fluidity

Methods S1: Low-molecular-weight organic acids

The HPLC system was equipped with a degasser, quaternary pump, autosampler, and a column thermostat set at 40°C. Separation of LMWOAs was achieved on a hydro-reversed-phase column Phenomenex Synergi 4 u Hydro-RP 80A, 250 × 3 mm^2^ combined with a guard column (4 × 2.0 mm^2^) of the same packaging material from Phenomenex (Torrance, California, USA) with 4 mM formic acid (solvent A) and MeOH (solvent B) as mobile phase. The LC system was routinely operated in a gradient mode and every sample was run with two chromatographic methods, which were found most sensitive for a number of selected LMWOAs. Thus, acetic, butyric, and oxalic acid were analysed with a post column addition of 1 M aq. ammonia (method I) and succinic, citric, lactic, D-gluconic and malic were analysed with the post column addition of methanol (method II). The chromatographic method I was performed using a gradient at a mobile phase flow rate of 0.2 ml min^-1^ starting with 84% A and 16% B for 10 min, followed by a linear gradient to 20% A and 80% B after 35 min and then returned to the initial conditions over 8 minutes. The method II was run with the mobile phase flow rate of 0.4 mL min^-1^ starting with 96% A and 4% B for 10 min, followed by a gradient up to 20% A and 80% B after 23 minutes and returning to the initial conditions over 7 minutes. Post column addition of 1 M aq. ammonia or methanol was performed by a micro pump (MPLC, model G, Brownlee Labs, San Francisco, CA, USA) set at 10% flow rate of the eluent, i.e., 20 µL min^-1^ or 40 µL min^-1^, respectively. For each chromatography experiment, 900 mL of sample was mixed with 100 mL of internal standard solution (2-furoic acid, 1 mM) and 50 µL of the resulting solution was injected. The single quadrupole MS was operated in the negative ionization mode. Monitoring of ions was carried out in the molecular mass range 50–500 Da to examine the fragmentation of organic acids. Quantification and identification of the separated analytes was done in the selected ion monitoring (SIM) mode primarily by matching the retention times of standards. Electrospray ioniziation (ESI) parameters were sequentially optimized via flow injection analysis using 50 µM standard solution of each LMWOA. The following parameters yielded highest signal intensities and were applied for all chromatographic runs: nebulizer gas (N_2_) 35 psi; drying gas (N_2_) flow rate 10 L min^-1^; capillary voltage 2500 V; gas temperature 350^o^C. The fragmentor voltage was set at 70 V. External standards within the range 0.01–1000 µM were used for calibration, and the data were processed using Agilent's Chemstation software package (Agilent Technologies Ireland Ltd., Cork, Ireland). Methanol (HPLC grade), formic acid (≥98%), and water (HPLC grade) were acquired from Carl Roth GmbH & Co (Karlsruhe, Germany). Acetic acid (≥99%), D-gluconic acid sodium salt (98%), oxalic acid (≥99%), and 2-furoic acid (98%) were acquired from Sigma-Aldrich (Steinheim, Germany). Citric acid monohydrate (extra pure), and DL-malic acid (>99.5%) were obtained from Merck (Darmstadt, Germany). Butyric acid (99%), lactic acid (98%), and succinic acid (99%) were acquired from Alfa Aesar GmbH & Co KG (Karlsruhe, Germany). Stock solutions of each standard (50 mM) were prepared in HPLC grade water and stored at -20^o^C until usage. For each P source, the mean concentration of each LMWOA was calculated for the periods where no P incorporation was detected in the AM plant tissue as well as for those where we detected P uptake in the AM plant.

Table S 1. Presence (%) of the different root traits for each treatment with access to a phosphorus source. Abbreviations are OP, orthophosphate; PA, phytic acid; GOE-PA, phytic acid bound to goethite; GOE-OP, orthophosphate bound to goethite.

| Root trait | | Arbuscules (%) | | | | | Vesicles (%) | | | | | Mycorrhization Rate (%) | | | | |
| --- | --- | --- | --- | --- | --- | --- | --- | --- | --- | --- | --- | --- | --- | --- | --- | --- |
| Day | | **21** | **35** | **49** | **77** | **91** | **21** | **35** | **49** | **77** | **91** | **21** | **35** | **49** | **77** | **91** |
| OP | Mean | 17.7 | 13.3 | 17.3 | 22.7 | 22.7 | 35.0 | 33.7 | 38.3 | 33.0 | 39.7 | 52.7 | 47.0 | 55.7 | 55.7 | 62.3 |
|  | se | 2.0 | 2.4 | 3.3 | 8.2 | 3.4 | 3.5 | 2.8 | 1.9 | 4.2 | 3.9 | 4.9 | 4.4 | 2.6 | 12.1 | 7.3 |
| PA | Mean | 8.0 | 12.3 | 20.0 | 14.0 | 17.3 | 15.3 | 37.0 | 33.7 | 36.7 | 36.7 | 23.3 | 49.3 | 53.7 | 50.7 | 54.0 |
|  | se | 1.2 | 3.3 | 4.0 | 1.2 | 3.7 | 0.3 | 10.0 | 8.5 | 2.9 | 2.7 | 0.3 | 13.3 | 12.3 | 2.7 | 5.9 |
| GOE-OP | Mean | 8.0 | 18.3 | 20.7 | 22.7 | 25.0 | 17.3 | 39.0 | 46.3 | 33.7 | 36.0 | 25.3 | 57.3 | 67.0 | 56.3 | 61.0 |
|  | se | 0.6 | 1.5 | 3.5 | 2.9 | 1.2 | 3.5 | 5.6 | 3.4 | 3.3 | 5.0 | 3.5 | 7.0 | 6.7 | 5.9 | 6.1 |
| GOE-PA | Mean | 11.7 | 12.0 | 20.3 | 22.0 | 20.0 | 19.3 | 25.7 | 32.0 | 46.7 | 33.3 | 31.0 | 37.7 | 52.3 | 68.7 | 53.3 |
|  | se | 2.0 | 3.1 | 3.4 | 4.6 | 1.0 | 2.3 | 7.8 | 4.0 | 3.3 | 3.2 | 4.0 | 10.8 | 6.7 | 7.8 | 2.9 |
